# Depressive symptoms and their gastrointestinal correlates: A nationwide observational study in the Chinese middle-aged and elderly population with nonmalignant gastrointestinal disorders

**DOI:** 10.1097/MD.0000000000048242

**Published:** 2026-04-10

**Authors:** Peng Cao, Yanfang Chen

**Affiliations:** a The First Affiliated Hospital, Department of Hepatopancreatobiliary Surgery, Hengyang Medical School, University of South China, Hengyang, Hunan, China; b The First Affiliated Hospital, Department of Neurology, Hengyang Medical School, University of South China, Hengyang, Hunan, China; c Clinical Research Center for Immune-Related Encephalopathy of Hunan Province, The First Affiliated Hospital, Hengyang Medical School, University of South China, Hengyang, Hunan, China.

**Keywords:** benign upper gastrointestinal disorders, China, depressive symptoms, middle-aged and older adults

## Abstract

The pathophysiological interplay between depressive symptoms and benign upper gastrointestinal (UGI) disorders encompassing peptic ulcer disease, gastritis, duodenitis, and gastroesophageal reflux disease remains incompletely defined. These chronic inflammatory conditions impose a substantial health burden on Chinese middle-aged and elderly adults, yet the temporal relationship linking depressive symptomatology to disease onset remains underexplored. This nationally representative cohort study seeks to address this critical knowledge gap through longitudinal analysis. This national population-based cohort study utilized China Health and Retirement Longitudinal Study data. Depressive symptoms were ascertained via the validated 10-item Center for Epidemiologic Studies Depression instrument; benign UGI disorders were defined as self-reported physician-diagnosed conditions. Multivariable-adjusted logistic regression models evaluated longitudinal associations. The longitudinal cohort comprised 4889 adults (48.4% female; mean age 59.6 ± 9.6 years). Adjusted analyses demonstrated dose-dependent associations: moderate and severe depressive symptoms conferred 1.419-fold (95% confidence interval 1.150–1.752) and 1.811-fold (95% confidence interval 1.021–3.035) elevated risks, respectively. Threshold analysis identified Center for Epidemiologic Studies Depression scores > 9 as clinically significant risk determinants (nonlinear *P* = .832). Elevated depressive symptomatology emerged as modifiable risk determinants for clinically significant risks of incident benign UGI disorders; a critical association particularly evident among community-dwelling Chinese adults (≥ 45 years).

## 1. Introduction

Benign upper gastrointestinal (UGI) disorders that comprise peptic ulcer disease, gastritis, duodenitis, and gastroesophageal reflux disease (GERD) are chronic inflammatory conditions with a multifactorial pathogenic basis.^[1,2]^ While Helicobacter pylori infection remains the cornerstone of current prevention strategies, growing evidence points to the involvement of psychosomatic factors in disease pathogenesis, a relevance accentuated by China’s simultaneous prevalence of population aging and late-life depression.^[3,4]^

Neurobiological models posit bidirectional gut-brain interactions through vagal neurotransmission, hypothalamic-pituitary-adrenal axis dysregulation, and cytokine-mediated pathways.^[5,6]^ Clinically, depressive symptomatology affects 32.1% of Chinese adults over 45, correlating with elevated risks of cardiometabolic and functional gastrointestinal comorbidities.^[4,7,8]^ Notably, existing research in this field has several critical limitations that our study aims to address: most studies focus on functional gastrointestinal disorders (FGIDs) or isolated GERD, with insufficient attention to benign UGI disorders encompassing organic pathologies (e.g., gastritis, peptic ulcers)^[9,10]^; cross-sectional designs, case-control studies, and genetic causal inference predominate,^[9,11,12]^ lacking longitudinal data to establish temporal relationships, especially in aging Asian cohorts^[12]^; few studies explore dose-response relationships between depression severity and incident UGI disorders, with most only verifying the presence of an association^[9,12]^; prior research has predominantly targeted young adults, Western populations, or convenience samples of psychiatric patients,^[9,11]^ with a dearth of nationally representative data from Chinese middle-aged and elderly populations.

This study innovatively examines the depression-UGI nexus through 3 analytical lenses: differential risk stratification by symptom severity, nonlinear threshold effects, and longitudinal confirmation in China’s aging population. Our findings address critical knowledge gaps in comorbidity management and preventive strategy development for organic gastrointestinal (GI) disorders.

## 2. Materials and methods

### 2.1. Study population

The China Health and Retirement Longitudinal Study (CHARLS) constitutes a nationally representative cohort employing multistage probability sampling across 28 provinces. Comprehensive details on the study design and methodology of CHARLS have been presented in prior publications.^[13]^ Our analytical sample derives from 17,705 baseline participants (wave 1), applying 3 exclusion criteria: age < 45 years (n = 391), prevalent UGI disorders (n = 1709), and incomplete core variables (n = 10716). Final analyses included 4889 adults with complete longitudinal data (2011–2015 waves).

Data for this study were from the CHARLS, which had obtained ethical approval from the Institutional Review Board of Peking University (IRB00001052-11015). During CHARLS’ baseline survey for database construction, all participants provided informed consent. As this study only involves secondary analysis of open-source CHARLS data, it qualifies for ethical exemption and adheres to relevant research ethics guidelines. Benign UGI disorders were operationalized as physician-diagnosed incident cases during follow-up. Depressive symptoms quantification utilized the 10-item Center for Epidemiologic Studies Depression (CES-D) scale, validated in Chinese populations.

**Figure 1.**
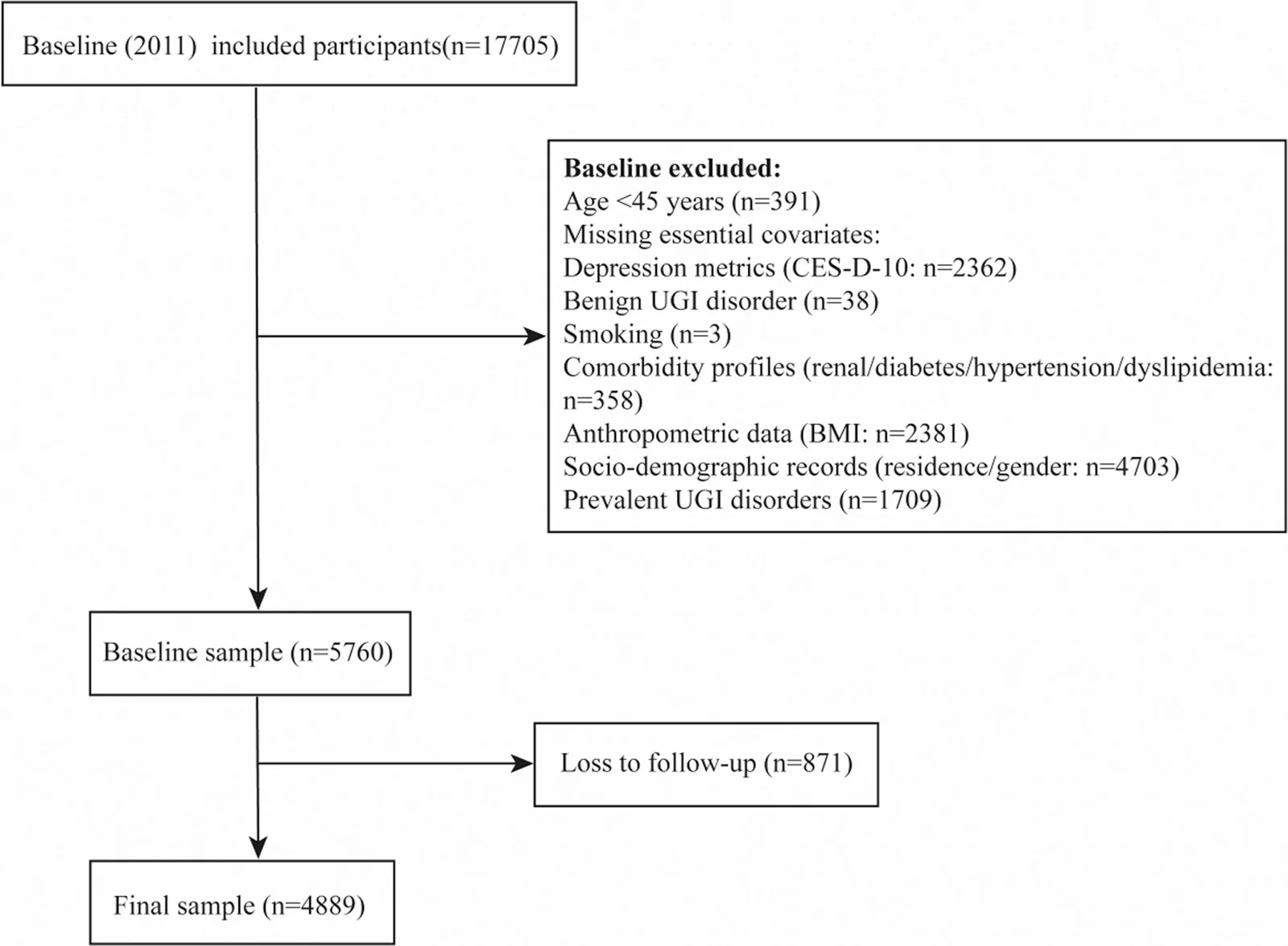
Flowchart for screening the sample population. BMI = body mass index, CES-D = Center for Epidemiologic Studies Depression, n = number of participants, UGI = upper gastrointestinal.

Covariate selection integrated demographic (age, gender, residence), anthropometric (body mass index [BMI]), and comorbidity parameters (hypertension, diabetes, dyslipidemia, renal disease). We implemented inverse probability weighting to address missing data bias. Statistical analyses progressed through 3 phases: baseline characteristic comparison using χ^2^/ANOVA tests, multivariable-adjusted logistic regression modeling, and restricted cubic spline (RCS) analysis for dose-response relationships. The sample selection process is outlined in detail in Figure 1.

## 3. Assessment of depressive symptoms

Depressive symptomatology was assessed and quantified using the Chinese-validated 10-item CES-D-10,^[14]^ a psychometrically robust instrument widely used in population studies of Chinese middle-aged and elderly adults, including the CHARLS.^[15,16]^ It shows robust internal consistency in CHARLS cohorts, with a reported Cronbach ɑ coefficient of 0.815 confirming reliability for this demographic.^[15]^

The CES-D-10 evaluates 3 core domains of depressive symptoms over a 7-day recall period, capturing clinically relevant manifestations: affective domain includes “I felt depressed” and “I felt lonely”; cognitive domain covers “I was bothered by things that usually don’t bother me” and “I had trouble concentrating”; somatic domain encompasses “My sleep was restless”, “Everything I did was an effort” and “I could not get going”. Two positively valenced items: “I felt happy” and “I felt hopeful about the future”, were included to reduce response bias and reverse-coded during data analysis.^[17]^

Each item uses a 4-tier Likert scale linked to symptom frequency: 0 = “rarely or none of the time ( < 1 day)”, 1 = “some or a little of the time (1–2 days)”, 2 = “occasionally or a moderate amount of the time (3–4 days)”, and 3 = “most or all of the time (5–7 days)”. Total scores range from 0 to 30, with clinically validated cutoffs for Chinese populations: none/mild (0–9), moderate (10–20), and severe (21–30). Consistent with prior CHARLS validation,^[17]^ a total score ≥ 10 indicated clinically significant depressive symptoms.

## 4. Assessment of benign UGI disorders

Benign UGI disorders targeted in this study include peptic ulcer disease, gastritis and duodenitis, and GERD. For operational clarity, these conditions were defined as self-reported cases confirmed by licensed physicians, with gastric, esophageal, and duodenal tumors systematically excluded as neoplastic lesions to ensure specificity of benign pathology classification.

Case ascertainment relied on the standardized health assessment questionnaire of the CHARLS. Participants were asked a structured UGI health query: “Have you ever been diagnosed with stomach or other UGI diseases excluding tumors or cancers by a physician?” All surveys were conducted by trained interviewers at baseline and each follow-up wave, following protocols aligned with international aging epidemiological studies to reduce assessment-related bias.^[18]^

For analytical consistency, a standardized dichotomous classification was used: participants confirming a physician diagnosis of any predefined benign UGI subtype were categorized as “present,” while those reporting no such diagnosis were classified as “absent.” This scheme was applied uniformly across follow-up to ensure temporal consistency in outcome definition, a practice validated in large-scale population cohorts.^[19]^

## 5. Assessment of other covariates

Patient demographics and clinical characteristics were systematically collected at baseline through standardized questionnaires. Demographic variables included age, gender, education level (illiterate, primary school and below, or middle school and above), residence (rural village vs an urban community), and marital status (married vs other, including never married, separated, divorced, or widowed). Anthropometric measurements were performed to calculate BMI using the formula weight (kg)/height^2^ (m^2^), which was categorized as normal (18.5–24 kg/m^2^), overweight (24–28 kg/m^2^), or obese ( ≥ 28 kg/m^2^). Lifestyle factors were assessed through smoking status (ex-smoker, nonsmoker, current smoker) and alcohol consumption (less than once a month, more than once a month, and none of these). Self-reported medical histories included hypertension, diabetes mellitus, chronic kidney disease, and dyslipidemia, all dichotomized as present/absent.

## 6. Statistical analysis

In the longitudinal cohort analysis, we stratified participants into 3 groups based on depressive symptom severity (none/mild, moderate, severe) as measured by the CES-D. Groupwise comparisons of baseline characteristics were performed using 1-way ANOVA/Kruskal-Wallis tests for continuous variables and chi-square tests for categorical variables. To examine the prospective association between depressive symptoms and benign UGI disorders at wave 3, we employed multivariable logistic regression modeling with 3 hierarchical specifications:

Crude model (Model 1): Unadjusted analysis of CES-D score against UGI outcomes.

Sociodemographic-adjusted model (Model 2): Additional covariates included age, sex, education level, residential status, and marital status.

Comprehensive adjusted model (Model 3): Further controlled for BMI, smoking status, alcohol consumption, hypertension, diabetes mellitus, dyslipidemia, and chronic kidney disease.

Interaction effects were assessed through product-term analysis (CES-D × covariate) to evaluate how sociodemographic factors, health behaviors, and anthropometric indices modify the CES-D-UGI relationship. RCS were applied to explore potential nonlinear associations and quantify dose-response relationships between CES-D severity and benign UGI disorders. All statistical analyses were conducted using R 4.3.3, with RCS implemented via the “regression modeling strategies" package. 2-tailed *P* values < 0.05 were considered statistically significant.

## 7. Results

### 7.1. Baseline characteristics

The cohort comprised 4889 participants (51.65% male; mean age 59.6 ± 9.6 years) with 417 incident cases of benign UGI disorders (8.52% cumulative incidence). Baseline depressive symptom burden, quantified by CES-D scores, averaged 9.7 ± 4.8 across the study population. Comparative analysis revealed distinct demographic patterning: individuals developing UGI disorders demonstrated younger age distribution (58.6 ± 9.7 vs 59.7 ± 9.6 years, *P* = .035), higher female predominance (56.83% vs 43.17%), and elevated depressive symptomatology (CES-D 10.8 ± 5.0 vs 9.6 ± 4.8) relative to unaffected counterparts (Table 1).

**Table 1:** Characteristics of the the participants at baseline (n = 4889).

	Overall	Without benign UGI disorders	With benign UGI disorders	*P*
	4889	4472	417	
Age	59.6 ± 9.6	59.7 ± 9.6	58.6 ± 9.7	.035
Gender				< .001
Female	2364 (48.35)	2127 (47.56)	237 (56.83)	
Male	2525 (51.65)	2345 (52.44)	180 (43.17)	
Education				.718
Illiterate	1199 (24.52)	1090 (24.37)	109 (26.14)	
Primary school and below	2046 (41.85)	1874 (41.91)	172 (41.25)	
Middle school and above	1644 (33.63)	1508 (33.72)	136 (32.61)	
Residence				.406
Rural village	3984 (81.49)	3651 (81.64)	333 (79.86)	
Urban community	905 (18.51)	821 (18.36)	84 (20.14)	
Marital status				.627
Married	1723 (35.24)	1571 (35.13)	152 (36.45)	
Other	3166 (64.76)	2901 (64.87)	265 (63.55)	
BMI	23.6 ± 4.0	23.6 ± 4.0	23.4 ± 3.5	.173
Smoking				.375
Ex-smoker	445 (9.10)	412 (9.21)	33 (7.91)	
nonsmoker	2815 (57.58)	2562 (57.29)	253 (60.67)	
Current Smoker	1629 (33.32)	1498 (33.50)	131 (31.41)	
Drinking				.208
Drink but less than once a month	386 (7.90)	352 (7.87)	34 (8.15)	
Drink more than once a month	1317 (26.94)	1220 (27.28)	97 (23.26)	
None of these	3186 (65.17)	2900 (64.85)	286 (68.59)	
Hypertension				.635
No	3723 (76.15)	3401 (76.05)	322 (77.22)	
Yes	1166 (23.85)	1071 (23.95)	95 (22.78)	
Diabetes				.997
No	4625 (94.60)	4230 (94.59)	395 (94.72)	
Yes	264 (5.40)	242 (5.41)	22 (5.28)	
Dyslipidemia				.085
No	4477 (91.57)	4105 (91.79)	372 (89.21)	
Yes	412 (8.43)	367 (8.21)	45 (10.79)	
Kidney disease				.078
No	4643 (94.97)	4255 (95.15)	388 (93.05)	
Yes	246 (5.03)	217 (4.85)	29 (6.95)	
CES-D (continuous)	9.7 ± 4.8	9.6 ± 4.8	10.8 ± 5.0	< .001
CES-D (categorized)				< .001
None/mild	2686 (54.94)	2497 (55.84)	189 (45.32)	
Moderate	2074 (42.42)	1863 (41.66)	211 (50.60)	
Severe	129 (2.64)	112 (2.50)	17 (4.08)	

Data are presented as mean ± standard deviations, number (proportion %).

BMI = body mass index, CES-D = Center for Epidemiologic Studies Depression, n = number of participants, UGI = upper gastrointestinal.

## 8. Threshold effects of CES-D scores on risk of benign UGI disorders

Table 2 delineates the graded association between depressive symptom severity and benign UGI disorders risk. We observed a significant monotonic trend across symptom severity strata (*P* for trend < .001). Compared to the none/mild symptom group, individuals with severe depressive symptoms demonstrated 1.81-fold elevated risk (odds ratio [OR] = 1.811, 95% confidence interval [CI] 1.021–3.035) after full adjustment for demographic and clinical covariates.

**Table 2:** Odds ratio (OR) and 95% CIs (confidence intervals) for the longitudinal relationship between depressive symptoms with benign upper gastrointestinal conditions: results from logistic regression model.

	Model 1	*P*	Model 2	*P*	Model 3	*P*
CES-D (continuous)	1.051 (1.030–1.071)	< .001	1.047 (1.026–1.069)	< .001	1.044 (1.023–1.066)	< .001
CES-D (categories)						
None/Mild	Ref		Ref		Ref	
Moderate	1.496 (1.219–1.838)	< .001	1.450 (1.177–1.788)	< .001	1.419 (1.150–1.752)	.001
Severe	2.005 (1.141–3.323)	.010	1.909 (1.080–3.188)	.018	1.811 (1.021–3.035)	.032
*P* for trend	< .001		< .001		< .001	

Model 1 was crude model. Model 2 was adjusted for age, gender, education, residence, and marital status. Model 3 was adjusted for age, gender, education, residence, marital status, BMI, smoking status, drinking status, hypertension, diabetes, dyslipidemia and kidney disease.

BMI = body mass index, CES-D = Center for Epidemiologic Studies Depression, CI = confidence interval, OR = odds ratio.

**Figure 2.**
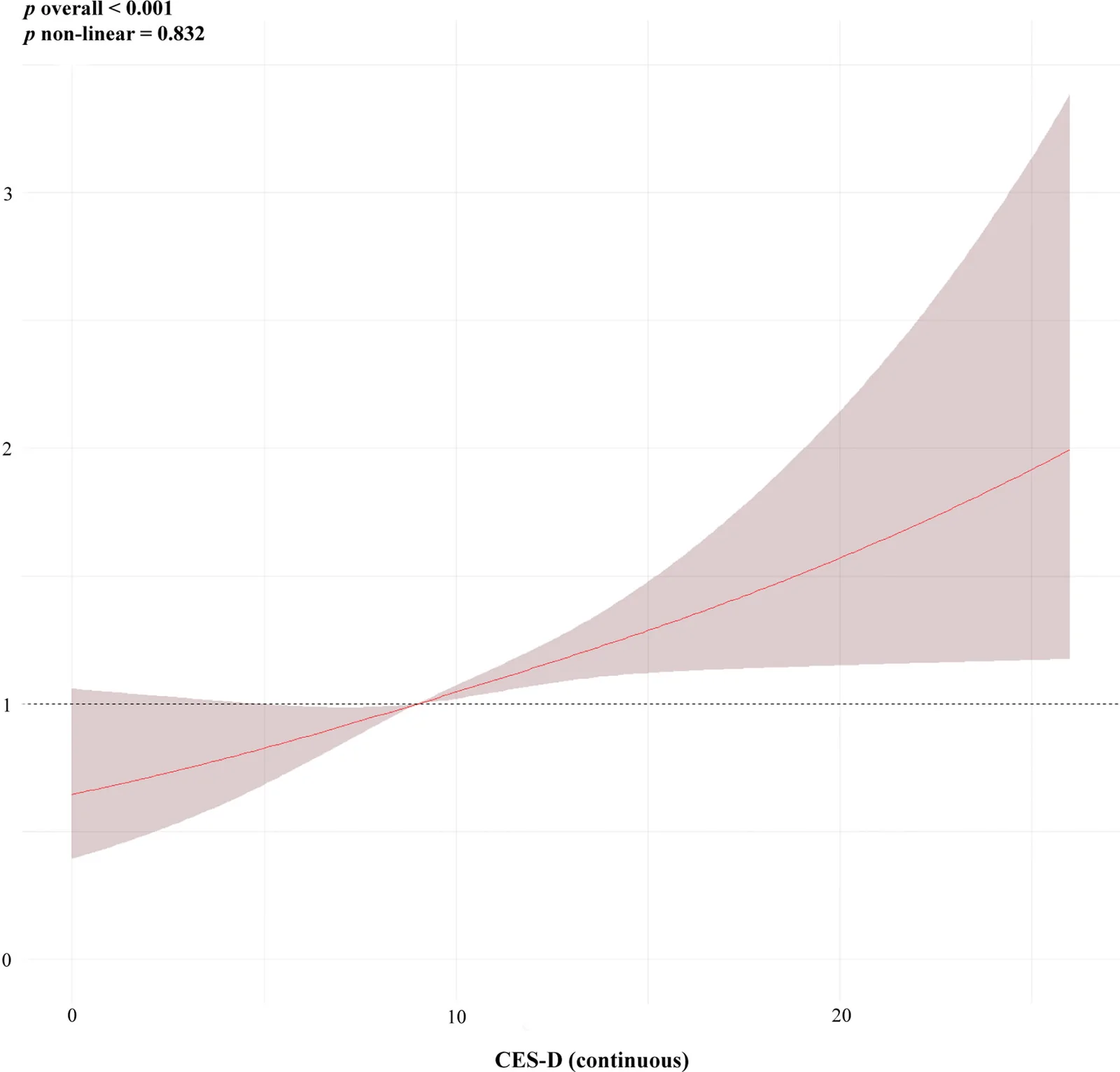
Restricted cubic spline of the association between CES-D and the risk of benign UGI conditions. The model was adjusted for age, gender, education, residence, marital status, smoking status, drinking status, BMI, hypertension, diabetes, dyslipidemia, and kidney disease. The figure does not indicate a significant nonlinear relationship between CES-D scores and the risk of benign UGI disorders. BMI = body mass index, CES-D = Center for Epidemiologic Studies Depression, UGI = upper gastrointestinal.

When analyzed as a continuous exposure, each unit increase in CES-D scores correlated with 4.4% risk elevation (OR = 1.044, 95% CI 1.023–1.066). The dose-response pattern displayed in Figure 2 maintained linear characteristics, with RCS analysis confirming absence of U-shaped association (*P*-nonlinear = .832).

## 9. Stratified analysis

**Figure 3.**
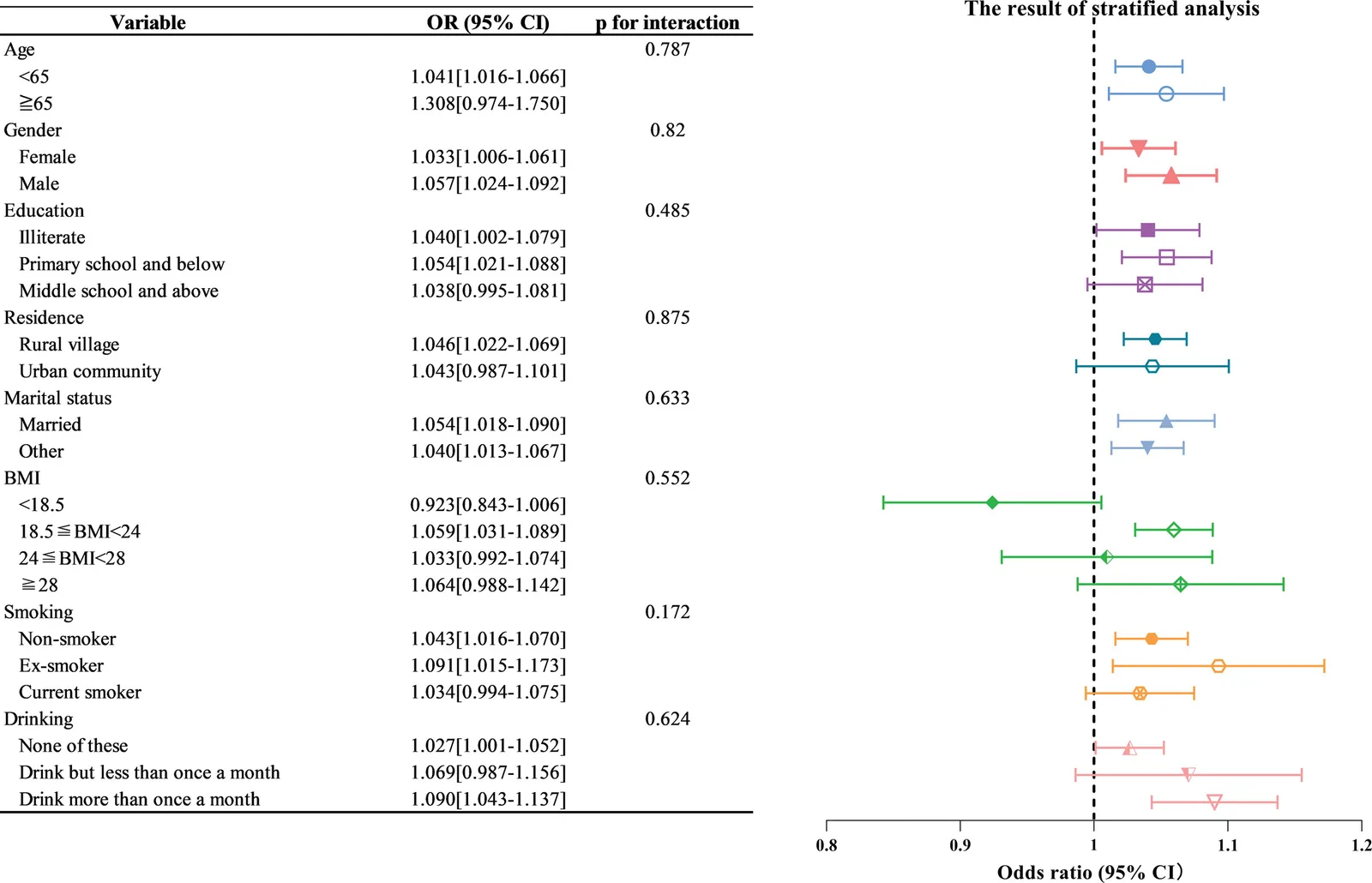
Forest plot of stratified analysis of the association of CES-D with the risk of benign UGI disorders. The plot indicates no notable interactions between CES-D and the other covariates. CES-D = Center for Epidemiologic Studies Depression, UGI = upper gastrointestinal.

Stratified analyses across demographic and clinical subgroups revealed homogeneous effect estimates of depressive symptomatology on UGI disorder risk. Multiplicative interaction terms between CES-D scores and covariates (age, gender, education level, cardiometabolic status) demonstrated nonsignificant effect modification (all *P* for interaction > .05). The consistent risk gradient persisted when assessing continuous CES-D exposure (Fig. 3).

## 10. Discussion

This prospective cohort study provides novel epidemiological evidence from China’s nationally representative CHARLS dataset, establishing temporality in the depression-UGI comorbidity relationship. Through longitudinal tracking with median 4 year follow-up, we demonstrated a graded risk elevation: individuals with severe depressive symptomatology exhibited 1.811-fold higher incidence of benign UGI disorders (95% CI 1.021–3.035) compared to the nondepressed reference group, persisting after full adjustment for metabolic and lifestyle confounders.

Emerging evidence from diverse study designs has advanced understanding of the depression-GI interplay, but key gaps remain. Cross-sectional studies have linked depression to FGID severity in young psychiatric outpatients^[9]^ and quantified associations between GI symptoms and late-life depression risk^[12]^; a Mendelian randomization study further confirmed genetic liability to depression as a causal factor for multiple GI diseases, including GERD and chronic gastritis^[11]^; and a mediation analysis highlighted the role of sleep disorders and somatic symptoms in the FGID-depression relationship.^[10]^ However, these studies either lack temporal clarity (cross-sectional designs),^[9,10,12]^ focus on genetic rather than clinical epidemiological associations,^[11]^ or omit organic benign UGI disorders and aging Chinese populations.^[9–12]^ In contrast, our nationwide longitudinal study fills these gaps by demonstrating that greater depression severity precedes incident benign UGI disorders in middle-aged and elderly Chinese adults, with a robust association persisting after adjusting for key confounders.

Notably, Jansson et al’s Norwegian population study revealed a synergistic effect of comorbid anxiety and depression on reflux risk.^[20]^ Li et al’s esophageal pH monitoring study in 518 GERD patients quantified depression severity associations with acid exposure parameters, with moderate-severe depression conferring 2.32-fold higher GERD prevalence.^[21]^ Our findings extend these observations by: establishing a temporal sequence (depression preceding UGI disease) that mitigates reverse causality concerns inherent to cross-sectional studies; expanding the disease scope to include organic benign UGI disorders rather than focusing solely on FGIDs or GERD; and providing data from a nationally representative Asian cohort, complementing Western and small-scale elderly studies.

The bidirectional gut-brain axis likely mediates the observed temporal association between depressive symptoms and benign UGI disorders.^[22]^ Chronic stress-induced hypothalamic-pituitary-adrenal axis dysregulation alters gut permeability and visceral sensitivity, potentially explaining the comorbidity pattern.^[23]^ Clinically, pro-inflammatory cytokines (e.g., IL-6, TNF-α) characteristic of depression exacerbate mucosal inflammation in functional dyspepsia and reflux esophagitis.^[24]^ Depressive symptoms also elevate UGI risk through behavioral pathways (physical inactivity, poor nutrition) and physiological mechanisms: stress-induced sympathetic/parasympathetic imbalance reduces lower esophageal sphincter pressure, disrupts motility, and increases gastric acid secretion.^[25,26]^ Immunologically, depression-associated reduced natural killer cell cytotoxicity, impaired IL-2 production, and CD8 T-cell dysfunction compromise Helicobacter pylori clearance, while gut microbiota alterations linked to depression further destabilize mucosal integrity.^[27–29]^

Notably, our adjusted (OR = 1.811) contrasts with both findings from a Chinese hospital-based cohort in Chengdu (2.32-fold elevations)^[21]^ and the genetic causal effect magnitudes reported in Mendelian randomization analyses,^[11]^ suggesting potential modulation by population characteristics or study design. This discrepancy arises from 2 distinct factors: first, our nationally representative sampling versus the Chengdu study’s single tertiary hospital cohort,^[21]^ which may introduce potential selection bias; second, Asian dietary patterns rich in fermented foods and cultural differences in somatization and symptom reporting: factors less explored in prior studies.^[9–12]^

Our study leveraged a nationwide cohort with longitudinal follow-up to investigate associations between depressive symptoms and benign UGI disorders. However, several methodological constraints warrant acknowledgment: the absence of granular clinical phenotypes for specific benign gastrointestinal pathologies precluded nuanced exploration of symptom-specific associations, potentially obscuring disease-specific mechanisms; our analysis failed to delineate distinct depressive symptom clusters or their pathophysiological mechanisms linked to UGI disorders, limiting insights into potential bidirectional pathways; retrospective self-reported assessments of benign UGI disorders introduced recall bias, which may have compromised the reliability of diagnostic accuracy; while prior evidence indicates that individuals with benign UGI disorders exhibit elevated depressive symptomatology,^[30,31]^ our design did not account for the bidirectional interplay between these conditions, potentially overlooking confounding effects from preexisting psychological vulnerabilities; the lack of severity metrics for benign UGI disorders prevented evaluation of dose-response relationships between symptom intensity and depressive manifestations, a critical gap given emerging evidence on symptom burden gradients.

## 11. Conclusion

Our study demonstrates a significant association between elevated depressive symptomatology and higher risk of benign UGI disorders in middle-aged and elderly Chinese populations. The observed correlation underscores the relationship between psychological health and GI disorders, suggesting that chronic psychosocial stress may contribute to mucosal barrier dysfunction through neuroendocrine pathways. These findings advocate for a paradigm shift in clinical practice, emphasizing the need to integrate targeted psychosocial interventions into routine gastroenterological care. Specifically, early identification of depressive symptoms through standardized screening tools and multidisciplinary management protocols combining pharmacotherapy with cognitive-behavioral strategies could potentially mitigate the progression of functional gastrointestinal disorders in at-risk populations.

## Acknowledgments

We are deeply grateful to all members of the CHARLS and every elderly survey participant for their invaluable time and energy voluntarily dedicated to this study.

## Author contributions

**Conceptualization:** Yanfang Chen.

**Data curation:** Peng Cao.

**Formal analysis:** Yanfang Chen.

**Funding acquisition:** Yanfang Chen.

**Investigation:** Peng Cao.

**Methodology:** Peng Cao.

**Software:** Peng Cao.

**Supervision:** Peng Cao, Yanfang Chen.

**Validation:** Peng Cao.

**Visualization:** Peng Cao.

**Writing – original draft:** Peng Cao.

**Writing – review & editing:** Yanfang Chen.
